# Identifying Areas with Low Access to the COVID-19 Vaccine: A New Objective Framework Incorporating Mobility Data

**DOI:** 10.3390/healthcare13121368

**Published:** 2025-06-06

**Authors:** Defeng Tao, Joseph Agor, Jessina McGregor, Trevor Douglass, Andrew Gibler, Hector A. Vergara

**Affiliations:** 1 School of Mechanical, Industrial, and Manufacturing Engineering, Oregon State University, 224 Rogers Hall, Corvallis, OR 97331, USA; hector.vergara@oregonstate.edu; 2 Johns Hopkins University Applied Physics Laboratory, 11100 Johns Hopkins Rd., Laurel, MD 20723, USA; joseph.agor@jhuapl.edu; 3 College of Pharmacy, Oregon State University, 1601 SW Jefferson Way, Corvallis, OR 97331, USA; mcgregje@oregonstate.edu; 4 Pharmacy and Policy Programs, Oregon Health Authority, 500 Summer Street NE, E-65, Salem, OR 97301, USA; trevor.douglass@oha.oregon.gov (T.D.);

**Keywords:** COVID-19, vaccines, health equity, spatial access, access disparities, access seasonality, racial disparities in access

## Abstract

Background: The disparities observed in COVID-19 vaccine access at the early stages of vaccine distribution highlight the need for vaccine distribution plans that consider equitable access. Strategies to identify areas with low access to vaccines that use a single pre-specified distance or time as a threshold to define accessibility may not represent reality. We propose a novel mobility data-driven (MDD) definition to identify areas that have low access to the COVID-19 vaccine. Methods: We collected geospatial mobility data for our MDD approach to determine areas of low access. We identified census tracts in Oregon with low access to the COVID-19 vaccine through two approaches—(1) an adapted United States Department of Agriculture (USDA) food desert definition and (2) our proposed MDD framework. Ten spatial and social measures of access were utilized to compare these two approaches. Results: Compered with USDA, low-access census tracts identified by the MDD definition have a lower spatial accessibility; higher rates of poverty, unemployment, uninsured individuals, and a population without high school diplomas; and a low per capita income. Moreover, we found that the proportion of older populations, as well as American Indian and Alaskan Native populations, as identified in the MDD low-access census tracts, is higher than that in the USDA definition. Conclusions: We believe that the new proposed framework using mobility data can identify more representative areas that have low access to COVID-19 vaccines. Our proposed framework provides a starting point for achieving the goal of the equitable distribution of resources.

## 1. Introduction

By 5 May 2023, when the World Health Organization (WHO) declared the end of the COVID-19 global health emergency, SARS-CoV-2 had infected more than 765 million individuals [1] and had taken about 6.93 million lives [2] since its emergence in 2019. Vaccinations significantly reduced the risk of hospitalization and mortality associated with COVID-19 [3]. However, during the early stages of vaccine distribution, supply limitations and access to the COVID-19 vaccine were significant issues facing public health entities. Supply limitations were prevalent even in the United States (U.S.), from when the first vaccine received emergency use authorization in December 2020 to the summer of 2021 when the supply of the vaccine increased [4,5]. Many COVID-19 vaccine distribution plans in the United States were created without input from health equity committees [6], which contributed to disparities in vaccination coverage among communities already experiencing higher rates of disease incidence and burden [7,8]. These disparities in COVID-19 vaccine access across different populations could have hindered progress toward reducing the negative effects of the pandemic [9]. Therefore, when distributing emergency resources like the COVID-19 vaccine, **equitable access** needs to be considered. We also acknowledge that an individual’s willingness to be vaccinated is also an important factor in preventing the spread of COVID-19 [10]. However, vaccine hesitancy is not within the scope of this paper.

People living in areas with low access to health and wellness services are commonly identified based on their geographic proximity to these services. The U.S. Department of Agriculture (USDA) has developed a methodology to identify areas with low access to food [11]. This definition has been previously adapted to identify areas that have low access to pharmacy services, as well as to define community pharmacy deserts [12,13,14]. More details will be discussed in Section 2. Another approach is to quantify spatial accessibility by combining the following two measures: (1) distance to the resource and (2) the ratio of supply-to-demand [15]. In this approach, the two-step floating catchment area method (2SFCA) [16] and its variants, such as the enhanced 2SFCA (E2SFCA) method [17] and the Gaussian 2SFCA (G2SFCA) method [18], have been widely utilized to evaluate healthcare accessibility [19,20].

In the approaches discussed above, a **threshold distance** or **threshold travel time** needs to be determined to identify whether an area is within a certain physical distance or travel time from a location. Methods to measure these thresholds have been attempted through dynamic parameter variation [21], guidelines provided by governmental entities [11,22], and survey-based approaches [23]. However, using those methods, the pre-specified numerical values are either subjectively determined by users [13,21] or are impacted by bias and subjectiveness in the survey design or approach [24]. Researchers have acknowledged that a conceptual framework for the analysis of these distances requires access to real world data [25]. Therefore, we argue that a more objective methodology is needed to more accurately identify populations with low access to vaccines and, more broadly, healthcare resources.

In this study, we propose and test a framework for a **mobility data-driven (MDD)** definition for identifying areas with low access to the COVID-19 vaccine. In this framework, we utilize mobility data to determine the threshold distance for rural and urban areas across different seasons. Travel patterns are significantly different in Oregon as weather patterns between seasons change drastically. To validate this approach, we apply an adapted USDA definition (as has been carried out in previous research) and a MDD definition to identify census tracts in Oregon that have low access to the COVID-19 vaccine. Then, we compare the accessibility of these low-access areas identified according to these two methods using 10 metrics.

The primary objective of this study is to demonstrate that a new framework driven by population mobility data to quantify threshold distances can provide a more robust representation of areas with low access to vaccines and healthcare resources. To the best of our knowledge, this is the first attempt to use mobility data as an objective measure of these distances to define vaccine accessibility. The rationale behind this approach is that mobility data, unlike other data sources, captures actual human behavior (i.e., where people travel and how far), thereby offering a more reliable indicator of willingness to travel and reducing the bias often introduced by subjective assumptions.

The rest of the paper is organized as follows. Section 2 describes the data sources, the adapted USDA definition, and our proposed framework (the MDD definition), along with 10 evaluation metrics. Section 3 presents the results from applying both the USDA and MDD definitions including the distribution of low-access census tracts in urban and rural areas, statistics on the calculated G2SFCA score and population-to-facility ratio, socioeconomic comparisons, and demographic distributions of the identified low-access areas. Section 4 discusses the insights derived from the comparison of our MDD approach with the adapted USDA definition in identifying areas in Oregon with low access to the COVID-19 vaccine. This section also addresses the limitations of our approach. Section 5 concludes the paper and presents areas for future work.

## 2. Materials and Methods

### 2.1. Population and Vaccine Provider Data

This study was conducted using population-level data from the state of Oregon, including 2625 non-zero US Census population block groups, which form 825 non-zero US Census population census tracts in Oregon. All demographic data at the census tract and block group levels were collected from the 2015–2019 American Community Survey (ACS) conducted by the U.S. Census Bureau [26]. More details can be found in Supplementary Table S1. The reason for using 5-year estimates of the ACS data for analysis is that the most recent 1-year estimate is not available for small geographic areas like census tracts and block groups. The longitude and latitude of all block groups were collected from TIGER/Line Shapefiles on the U.S. Census Bureau website [27].

The urbanicity of census tracts or ZIP codes is determined by the U.S. Department of Agriculture Rural-Urban Commuting Area (RUCA) code standard [28]. The classification of ten categories can be found in Supplementary Table S2. In Oregon, a census tract or ZIP code is defined as being rural if its RUCA code is 4 or greater based on the definition provided by the Oregon Office of Rural Health [29].

We obtained the names and addresses of all approved COVID-19 vaccine providers from the Oregon Health Authority [30]. A total of 1384 unique vaccination sites in Oregon were identified, including pharmacies and other healthcare sites. Using the physical address of each vaccination site registered with the state, the longitude and latitude of their physical location were obtained using ArcGIS Pro 3.0.

We conservatively estimate that each vaccine provider has a daily capacity to administer 32 vaccinations, based on an estimated rate of four vaccinations per hour over an eight-hour day. This estimate reflects the typical capacity of a community retail chain or supermarket-based pharmacy in the U.S., where pharmacists provided for approximately half of all initial COVID-19 vaccinations [31]. The Oregon Board of Pharmacy requires that during operating hours, at least one pharmacist is present. During the early phases of COVID-19 vaccine distribution, appointments were required at pharmacy settings, and major retail chain- and supermarket-based community pharmacies scheduled appointments in 15 min intervals. In addition, the U.S. Centers for Disease Control and Prevention recommends a 15 min post-vaccination observation period [32]. Thus, we estimate that a pharmacy can vaccinate four people per hour. Based on the historical flu vaccination data from Oregon state, an average of 23–26 flu vaccines are provided per day during peak season [30]. However, regulatory changes were made to expand pharmacy abilities to administer the COVID-19 vaccine during the pandemic [31], and more vaccines were administered. Using these assumptions, we estimate that all vaccination sites combined could administer approximately 310 K doses per week. This figure closely aligns with the Oregon Health Authority’s early-stage target of 320 K doses per week [33].

### 2.2. Road Network Data

Vehicle travel time between each population (block group) and vaccination site was collected using the Open Source Routing Machine (OSRM) [34], which is a library combining routing algorithms and OpenStreetMap road network data.

### 2.3. SafeGraph Mobility Data

Society increasingly relies on location-based services such as GPS navigation and ridesharing apps for everyday travel decisions. SafeGraph, which is a U.S.-based commercial company, provides location-based service data and point-of-interest (POI) data across North America [35]. A POI refers to a physical location that individuals may visit for recreation or necessity (e.g., an amusement park, grocery store, or school). SafeGraph’s visit pattern data (a type of location-based service data) offer insights into when individuals (identified via their mobile devices) arrive at various POIs, how long they stay, and how far they travel from home [36]. This dataset tracks the same POIs and individuals over time, and prior research has shown it to be a reliable representation of population movement [37]. As a result, it has been widely used in various COVID-19-related studies [38,39,40].

For this study, we collected and analyzed SafeGraph visit patterns data for specified points-of-interest (POIs) across Oregon from 1 March 2019 to 28 February 2020. We indicate this period as the period before the COVID-19 pandemic started in Oregon and utilize mobility during this period to capture people’s “normal” behavior as it pertains to seeking healthcare services. Three North American Industry Classification System (NAICS) codes were selected to reflect regular healthcare services and determine a threshold distance by the median distance from home traveled by visitors to the POIs with the following three codes: 456,110—Pharmacies and Drug Stores; 6211—Offices of Physicians; and 6221—General Medical and Surgical Hospitals [41].

### 2.4. Adapted USDA Low-Access Definition

The USDA approach was originally developed to identify areas with limited access to food, commonly referred to as food deserts [11]. Due to the similarity in addressing issues of access to essential services in underserved communities, Qato et al. [13] were the first to adapt the USDA methodology to identify community pharmacy deserts in Chicago. Since then, this adapted definition has been widely applied in other studies to identify pharmacy deserts in various regions [12,14].

The USDA definition identifies low-access food areas at the census tract level using two approaches, as explained in Supplementary Table S3. In this study, we adopted Approach 1, which used the third threshold distance to identify census tracts with low access to vaccines by substituting “vaccine provider” for “supermarket”. Specifically, we considered a census tract to have low access to the COVID-19 vaccine if more than 500 people or 33% of the population of that tract live further than the threshold distance (1.61 km and 32.19 km for urban and rural areas, respectively) from the nearest vaccine provider.

### 2.5. Mobility Data-Driven Definition

We adopted a similar mindset as the USDA for our proposed framework. Namely, an area was considered to have low access if a significant share of individuals lived further than a threshold distance from the vaccination site. However, the key difference in our approach is that we utilized mobility data to determine these components through two sequential steps—step (1): threshold distances determination; step (2): significant share proportion determination.

In our initial analysis of the USDA’s interpretation of “significant share” (i.e., more than 500 people or 33% of the population), we found that some census tracts were classified as “low access” because more than 500 people were outside the threshold distance of the nearest vaccine provider. However, the equivalent proportion of some tracts was significantly lower than 33% (e.g., 600 people out of 8000). As a result, this can potentially lead to identifying a large proportion of individuals as having low access when, in reality, they are within the threshold distance of a vaccine provider. Thus, instead of directly determining the significant share, we proposed a way to determine it by percentile.

Step 1: Calculate the threshold distance

The SafeGraph mobility data provide two elements that we use for determining the threshold distances—(a) median distance from home to POIs and (b) the ZIP code where a POI is located. To control the effects of seasonality, we divide the data into four seasonal time periods—spring (March–May), summer (June–August), fall (September–November), and winter (December–February). For each season, we select the POIs representing regular healthcare service locations and categorize them as either a rural (category = r) or urban (category = u) POI based on its ZIP code. We utilized a commonly accepted non-parametric method identifying the upper limit for outliers in data [42] to represent the limit of people’s willingness to travel, i.e., the threshold distance, as follows:

(1) $$d_{s}^{k}=d_{Q3}+1.5\times \left(d_{Q3}-d_{Q1}\right)$$

where $d_{s}^{k}$ is the threshold distance for POI category k (r or u) in season s; $d_{Q1}$ and $d_{Q3}$ are the first and third quartiles of the collection of median distances from home to provider locations of type k in season s, respectively.

Step 2: Determine the “significant share” of a population

First, we identify each census tract that has at least one block group within its region with no access to the vaccine (for each season). In order to keep consistent with SafeGraph data, we calculate the Haversine distance [43] between the centroid point of block group p and a vaccine provider f, denoted as $d_{pf}$, as follows:

(2) $$d_{pf}=2R\times arcsin\left(\sqrt{{sin}^{2}\left(\frac{{lat}_{p}-{lat}_{f}}{2}\right)+cos({lat}_{p})\cos({lat}_{f}){sin}^{2}\left(\frac{{lon}_{p}-{lon}_{f}}{2}\right)}\right)$$

where R is the radius of the earth, 6371.38 km; ${lon}_{f}$ and ${lat}_{f}$ are the longitude and latitude of the vaccine provider f; and ${lon}_{p}$ and ${lat}_{p}$ are the longitude and latitude of block group p.

A block group p is considered to have no access to the COVID-19 vaccine if in the current season s, its distance $d_{pf^{\prime }}$ to the nearest vaccine provider $f^{\prime }$ (which is of type k) is greater than the threshold distance $d_{s}^{k}$. Using the population of each block group gathered from the US Census Bureau data, we calculate the proportion of each census tract that had no access. Finally, amongst the four different proportions calculated for each season, we utilize the smallest 25th percentile of these calculated values, denoted as Γ, as the desired **significant share** proportion.

**Figure 1:**
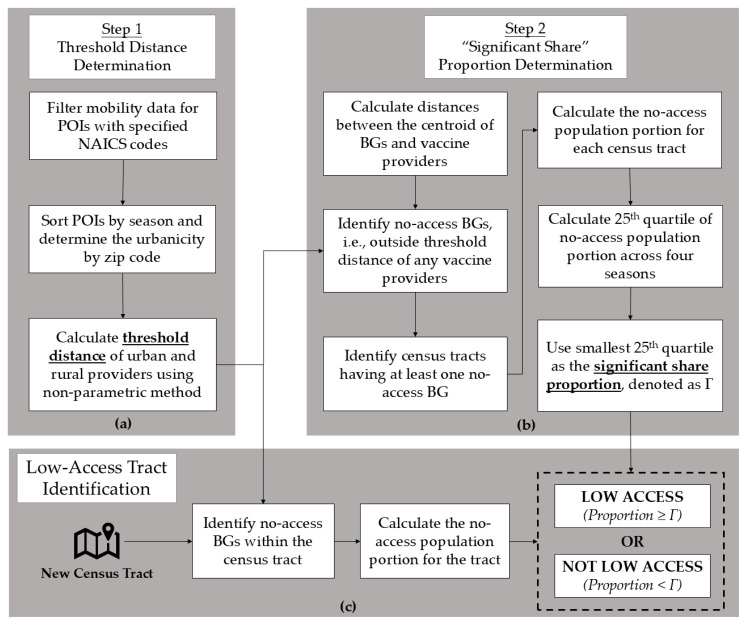
Proposed mobility data-driven (MDD) framework for the identification of areas with low access to the COVID-19 vaccine. Subfigure (**a**): In each season, threshold distance is determined for rural and urban areas. Subfigure (**b**): Significant share proportion determination for all seasons. Subfigure (**c**): Low-access census tracts identification for each season.

Therefore, a census tract is defined to have low access to the COVID-19 vaccine if the proportion of the population of that census tract that has no access to the vaccine is larger than or equal to Γ. Figure 1 provides an overview of our proposed MDD framework for the identification of areas with low access to the COVID-19 vaccine. We also performed a sensitivity analysis between the 5th percentile and 30th percentile with 5 percentile increments and the situation that Γ is 0, i.e., a census tract is considered to be low access as long as it contains a no-access block group. 

### 2.6. Evaluation Metrics

To evaluate the efficacy of our proposed framework, we utilized both spatial and social measures as evaluation metrics. G2SFCA is an approach that is widely adapted to evaluate healthcare accessibility [18]. A detailed introduction to the G2SFCA approach and sample examples can be found in the Supplementary Spatial Evaluation Metrics Section. To calculate this score, a threshold distance or travel time, $d_{0},$ needs to be defined. For testing purposes, we do not utilize USDAs or our threshold distances for this $d_{0}$ value as this will introduce bias towards the respective method. Therefore, we vary the threshold of travel time by between 20 min and 90 min, in 10 min increments, and calculate the accessibility score for each time.

However, the G2FSCA accessibility score can be difficult to interpret or understand from a practical standpoint. Namely, given a score for a census tract, it can be challenging to understand what the magnitude of that score means as it relates to access and how it can be translated to what actions should be taken, if any. The population-to-facility ratio of a census tract represents, on average, the number of individuals served by a single provider. The reasoning behind using this as an evaluation metric is that the units of measurement of this metric make it easier for a user to understand changes in its value. Namely, a unit increase in this measure implies that another individual is being served by a single provider within that area, on average. The more people that are served by a single provider, the less access there is to that provider due to the amount of work they are experiencing. Hence, the larger the population-to-facility ratio is in an area, the less access that population has. Examples can also be found in the Spatial Evaluation Metrics Section of the Supplementary Materials.

Socioeconomic status includes five indicators—poverty rate, unemployment rate, the proportion of the tract that has no high school diploma, the percentage of the tract that is uninsured, and the per capita income of the tract. Age is categorized into four groups—under 5, 5 to 19, 20 to 64, and 65+ years of age. The categories for race include White, Black or African American, American Indian and Alaska Native, Asian, Native Hawaiian and Pacific Islander, other, and two or more races. Ethnicity includes Hispanic or Latino and Non-Hispanic or Latino. The reason we include races and ethnicity is that multiple studies have shown that in the U.S., there are racial and ethnic disparities in COVID-19-related infection and death [44,45,46], as well as vaccination rate [47]. The overview of the comparison process can be found in Supplementary Figure S2.

## 3. Results

**Figure 2:**
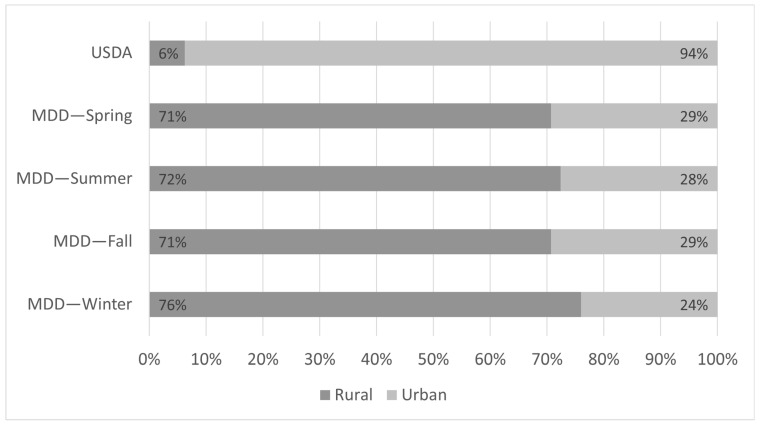
Rural and urban distribution of low-access census tracts identified by the MDD and United States Department of Agriculture (USDA) frameworks.

**Figure 3:**
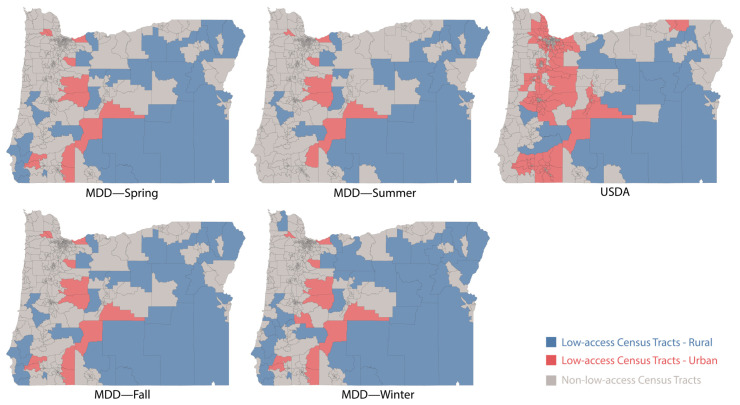
Rural and urban distribution comparison of low-access census tracts in Oregon; Γ = 20.84% for the 25th percentile.

Using the adapted USDA definition, 272 census tracts are defined as having low access to the COVID-19 vaccine. Among these, 17 and 255 are classified as rural and urban, respectively. Using our MDD framework, we determined the threshold distances (in km) for rural areas as 24.15, 27.50, 24.27, and 22.01 in spring, summer, fall, and winter, respectively. For urban areas, we determined these distances (in km) to be 20.27, 21.61, 20.49, and 21.01 in spring, summer, fall, and winter, respectively. The significant share, Γ, of the population was calculated as 20.84% for the 25th percentile. Applying this definition, we identify 41 (29 rural and 12 urban), 29 (21 rural and 8 urban), 41 (29 rural and 12 urban), and 50 (38 rural and 12 urban) census tracts as having low access to the COVID-19 vaccine in the spring, summer, fall, and winter seasons, respectively. Figure 2 provides a summary of these distributions, while Figure 3, which was generated by Tableau Public, depicts this distribution geographically. Compared with the threshold distances in all seasons, we noticed that for some census block groups, individuals may need to travel more than 50 km, with the maximum distance being 119 km, to reach the nearest vaccine provider.

For both the adapted USDA and MDD definitions, we calculate the G2SFCA accessibility score and population-to-facility ratio for each census tract identified as having low access, varying the travel time thresholds ($d_{0}$) between 20 and 90 min with 10 min increments. Table 1 provides the median and interquartile range among all low-access census tracts across all travel time thresholds. The median G2SFCA score ranges from 0 to 0.15, 0 to 0.09, 0 to 0.15, and 0 to 0.16 for spring, summer, fall and winter, respectively. These are also lower than those identified by the USDA definition (ranging from 0.24 to 0.35) across all threshold travel times. The median population-to-facility ratio ranges from 60 to 4954, 66 to 4954, 60 to 4954, and 50 to 4954 for spring, summer, fall and winter, respectively. These are also lower than those identified by the USDA definition (ranging from 4 to 50) across all threshold travel times.

The median rates of poverty, unemployment, lack of high-school diploma, and uninsured population of low-access census tracts identified according to the MDD definition across all seasons are higher than those of the low-access census tracts identified according to the adapted USDA definition. The median per capita income of low-access census tracts identified by the MDD definition across all seasons is lower than those identified by the adapted USDA definition.

Age and race distributions are provided in Table 2. Notice that within the census tracts identified as having low access by both the adapted USDA definition and our proposed MDD definition, the proportion of those aged 65 years and older is higher than the state’s distribution. Although this is expected since both definitions identify low-access areas, we further observe that the proportion is much higher when utilizing our MDD definition across all seasons. When comparing race, we notice similar patterns of White populations and American Indian and Alaskan Native populations on tracts identified by the adapted USDA definition and our proposed MDD definition. However, the proportion of American Indian and Alaskan Native populations is much larger amongst those census tracts that were identified by the MDD framework. Moreover, we notice that this proportion among those identified by the adapted USDA definition is lower than the state’s proportion.

Sensitivity analysis results can be found in the Supplementary Sensitivity Analysis Section. The number of identified low-access census tracts decreases when the significant share of individuals living further than a threshold distance from the vaccination site increases. However, the results of the metrics comparison are not sensitive to significant share change. From the 5th percentile to the 30th percentile, or even when Γ is equal to 0, the metrics always indicate that our MDD definition identifies more representative areas that have low access to COVID-19 vaccines.

## 4. Discussion

This study demonstrates that a mobility-based framework can identify a more focused set of population blocks of people with low access to vaccines and that these low-access groups have a higher proportion of people greater than 65 years of age and of American Indian/Alaska Native race compared to the USDA-based set of low-access population blocks. This is advantageous as it can be used to better target limited public health resources for those with the greatest need.

In our MDD framework, instead of determining the threshold distances arbitrarily, we calculate the threshold distance for each season based on people’s observed mobility data. The calculated urban area threshold distance ranged from 20.26 to 21.60 km, which is considerably larger than the 1.61 km distance used in the adapted USDA definition. This is concordant with prior research reporting that people residing in urban areas travel much more than 1.61 km to receive healthcare services [48,49]. Similarly, for rural areas, the adapted USDA definition could have utilized 16.09 or 32.19 km as a threshold distance (in our analysis, we implemented 32.19 km). Our MDD framework yielded a threshold distance for rural areas ranging from 22.01 km to 27.50 km. Multiple previous studies [49,50] indicate that the threshold distance for rural areas varies drastically depending on the rural area, and it is unreasonable to assign an arbitrary threshold number to all rural areas.

One strength of our study is considering seasonality in travel patterns, which is especially important in areas experiencing drastic changes in weather patterns between seasons. In our analysis, we find that people living in rural areas tend to travel further during the summer to receive healthcare services, but less in winter due to the poorer driving conditions resulting from harsher weather. People in urban areas of Oregon have similar travel patterns during the spring, summer, and fall seasons. However, the threshold distance for urban areas during winter is higher than that in the spring and fall. Previous research indicates that the spread of infectious diseases, especially influenza, occurs during the winter season [51], and the high population density in urban areas plays a key role in this spread [52,53]. Therefore, one can hypothesize that due to higher levels of infection in high-density areas, there will be more people accessing healthcare services. However, due to the lack of an increase in providers or pharmacy locations, these individuals may be more willing to travel further due to the increased number of visits under the same number of resources. In addition, in our analysis, the threshold distances in spring and fall are very close for both rural and urban areas, which leads to the identified low-access census tracts and metrics comparison results being identical in these two seasons.

We observed that the census tracts identified by our MDD framework have lower median G2SFCA accessibility scores and higher median population-to-facility ratios. This indicates that individuals residing in the low-access census tracts identified by our framework have less accessibility to the vaccine via these metrics. Furthermore, we also found that our MDD framework identified census tracts with a population with higher poverty rates, higher unemployment rates, a higher proportion of individuals without a high school diploma, a larger proportion of individuals who are uninsured, and a lower per capita income, on average. This is consistent with prior studies showing that individuals or households with a lower socioeconomic status were less likely to be vaccinated during the COVID-19 pandemic [54,55,56].

With respect to race and ethnicity distribution, we observed that several minority groups such as Black or African American, Asian, Native Hawaiian and Other Pacific Islander, individuals of some other races, and those identifying with two or more races make up a smaller proportion of the population living in low-access census tracts identified by the MDD definition compared to those identified by the USDA definition and the overall population of Oregon. However, two racial groups, White and American Indian/Alaska Native, are over-represented in the low-access census tracts identified by the MDD framework relative to their proportions in the overall state population of Oregon. In the case of the White population, we suggest that this reflects an access issue driven by poverty rather than race. As previously noted, studies have shown that individuals with lower incomes (regardless of race) face greater barriers to healthcare access, including lack of insurance, limited transportation, unaffordable childcare, and difficulty taking time off work. Our findings support this interpretation that the average per capita income for White individuals living in low-access census tracts identified by the MDD definition for each season (ranging between USD 30,025 and USD 30,561) is lower than that of White individuals in low-access census tracts identified by the adapted USDA definition (USD 37,286) and the statewide average for Oregon (USD 35,429). As for the American Indian and Alaska Native populations, these communities were disproportionately affected by COVID-19 and experienced lower vaccination rates [47,57]. Compared to the adapted USDA definition, our proposed MDD framework captures this disparity by identifying regions that have larger proportions of populations with low levels of access to the COVID-19 vaccine.

In the adapted USDA definition, the identified low-access urban census tracts represent a large portion of all low-access census tracts (255 out of 272) and disproportionally affect values of metrics, as shown in the Supplementary USDA’s Metrics—Urban and Rural. In our MDD definition, 20 to 48 rural and 7 to 14 urban census tracts are identified as having low access in different seasons as different significant shares of individuals lived further than a threshold distance from the vaccination site, as shown in the Supplementary Table S5. Although selecting different significant shares changes the number of identified low-access census tracts or the number of identified urban census tracts, the change is minimal when comparing it to the number of USDA-identified low-access urban census tracts. Therefore, comparison results are not sensitive to the change in significant share.

There are also some limitations to our research. Previous research [58] using administrative data to audit potential bias in the use of mobility data has suggested that the mobility of older and non-white voters might be less represented by SafeGraph data compared to voter roll data. However, mobility pertaining to voting is different to healthcare mobility data. Future research is needed to evaluate the best resources for evaluating the mobility of specific sub-populations. In addition, there were approximately 20% missing values for the median travel distance from home for each month in Oregon, from March 2019 to February 2020. In this work, we have excluded these POIs from our analysis. Furthermore, while our estimate of 32 vaccines per day is reasonable given the available data and knowledge of pharmacy operations, in reality, the actual capacity to administer vaccines varies according to vaccination site. Further research is needed to test our approach in comparison to other varied approaches to account for capacity. 

## 5. Conclusions

We propose a mobility data-driven framework to identify areas that have low access to healthcare resources like COVID-19 vaccines. This framework adapts the USDA food desert definition but also introduces a method that incorporates mobility data to determine threshold distances that define access. We validated the use of this framework by comparing it to an adapted USDA definition, which is what has been conducted in previous research on healthcare access. Overall, we find that the use of our proposed framework can identify areas that are more representative of those that truly have low access to the COVID-19 vaccine.

We believe this study serves as a valuable starting point for exploring the integration of mobility data into efforts to promote healthcare access equity. While the data presented in this study specifically reflect the identification of areas with low access to COVID-19 vaccination in the state of Oregon, the proposed framework is broadly applicable. It can be adapted to various contexts where identifying low-access areas to essential resources is necessary and can be extended to regions that include both urban and rural communities. Therefore, we recommend incorporating mobility data into future planning efforts for the equitable distribution of emergency resources, as it offers a more objective and behavior-based understanding of accessibility.

## Figures and Tables

**Table 1:** Comparison of Gaussian two-step floating catchment area (G2SFCA) method score, population-to-facility ratio, and socioeconomic status in areas with low access to the COVID-19 vaccine in Oregon identified by two different frameworks; Γ = 20.84% for the 25th percentile. The bold numbers represent median values indicating lower access compared with the USDA.

Metrics		MDD—Spring		MDD—Summer		MDD—Fall		MDD—Winter		USDA	
		MED	IQR	MED	IQR	MED	IQR	MED	IQR	MED	IQR
Calculated Metrics	$d_{0}$										
G2SFCA score	20	**0.00**	0.08	**0.00**	0.02	**0.00**	0.08	**0.00**	0.15	0.24	0.23
	30	**0.00**	0.13	**0.00**	0.09	**0.00**	0.13	**0.01**	0.19	0.25	0.24
	40	**0.04**	0.14	**0.02**	0.09	**0.04**	0.14	**0.04**	0.17	0.29	0.23
	50	**0.05**	0.14	**0.03**	0.08	**0.05**	0.14	**0.06**	0.19	0.32	0.22
	60	**0.08**	0.15	**0.04**	0.11	**0.08**	0.15	**0.09**	0.18	0.33	0.19
	70	**0.09**	0.17	**0.06**	0.13	**0.09**	0.17	**0.11**	0.18	0.33	0.16
	80	**0.12**	0.17	**0.09**	0.15	**0.12**	0.17	**0.13**	0.17	0.34	0.13
	90	**0.15**	0.16	**0.09**	0.13	**0.15**	0.16	**0.16**	0.16	0.35	0.11
Population-to-facility ratio	20	**4954**	4133	**4954**	3815	**4954**	4133	**4954**	4176	50	243
	30	**4954**	4653	**4954**	4648	**4954**	4653	**1386**	4647	23	65
	40	**449**	2258	**472**	2295	**449**	2258	**450**	2290	14	31
	50	**260**	1498	**413**	1506	**260**	1498	**252**	1365	11	20
	60	**151**	3180	**181**	3176	**151**	3180	**143**	896	8	15
	70	**122**	919	**122**	2417	**122**	919	**111**	399	6	12
	80	**91**	306	**106**	451	**91**	306	**75**	234	5	10
	90	**60**	223	**66**	305	**60**	223	**50**	189	4	9
Non-calculated Metrics	Indicator										
Socioeconomic Status	Poverty Rate (%)	**13.30**	8.20	**13.00**	7.10	**13.30**	8.20	**13.50**	8.03	9.65	8.00
	No High School Diploma Rate (%)	**8.56**	4.29	**8.39**	3.69	**8.56**	4.29	**8.66**	4.60	7.59	6.70
	Unemployment Rate (%)	**6.80**	4.60	**6.60**	4.80	**6.80**	4.60	**6.80**	4.48	4.90	3.60
	Uninsured Rate (%)	**6.50**	2.80	**6.50**	3.10	**6.50**	2.80	**6.50**	2.73	5.65	4.10
	Per Capita Income	**27.5 K**	7.7 K	**28.7 K**	8.5 K	**27.6 K**	7.7 K	**27.6 K**	7.3 K	34.0 K	13.9 K

**Table 2:** Frequency of age, race, and ethnicity distribution in Oregon versus areas with low access to vaccines. The bold numbers represent values higher than numbers in All Oregon column.

	All Oregon	Low-Vaccine-Accessibility Areas by Definition				
		USDA	MDD—Spring	MDD—Summer	MDD—Fall	MDD—Winter
Age (years)						
Under 5	5.58%	5.49%	4.45%	4.66%	4.45%	4.56%
5 to 19	17.80%	**18.51%**	15.60%	16.55%	15.60%	15.57%
20 to 64	59.43%	57.29%	54.58%	54.70%	54.58%	54.41%
65+	17.18%	**18.71%**	**25.37%**	**24.09%**	**25.37%**	**25.47%**
Race						
White alone	84.29%	**86.51%**	**88.77%**	**87.83%**	**88.77%**	**89.23%**
Black or African American alone	1.91%	1.24%	0.71%	0.78%	0.71%	0.62%
American Indian and Alaska Native alone	1.16%	0.96%	**4.50%**	**5.74%**	**4.50%**	**4.28%**
Asian alone	4.37%	4.01%	0.78%	0.76%	0.78%	0.69%
Native Hawaiian and Other Pacific Islander alone	0.40%	0.29%	0.14%	0.14%	0.14%	0.11%
Some other race alone	3.07%	2.52%	1.27%	1.44%	1.27%	1.11%
Two or more races	4.80%	4.47%	3.84%	3.31%	3.84%	3.95%
Ethnicity						
Hispanic or Latino	13.01%	11.25%	7.25%	7.45%	7.25%	6.76%
Non-Hispanic or Latino	86.99%	**88.75%**	**92.75%**	**92.55%**	**92.75%**	**93.24%**

## Data Availability

The data generated or analyzed are from the Oregon Health Authority and are not publicly available but can be made available from the corresponding author on reasonable request.

## Supplementary Materials

The following supporting information can be downloaded at https://www.mdpi.com/article/10.3390/healthcare13121368/s1, Table S1: Demographic data collected from the United States Census Bureau; Table S2: U.S. Department of Agriculture Rural-Urban Commuting Area (RUCA) code; Table S3: USDA low access definition (food). Spatial Evaluation Metrics Section—Figure S1: G2SFCA accessibility calculations example; Figure S2: Overview of the research process. Sensitivity Analysis Section—Table S4: Significant share determined by percentile; Table S5: Rural and urban distribution of low-access census tracts; Figures S3–S8: Geographic distribution of identified low-access census tracts across seasons with comparison of USDA in Oregon when Γ is from 0 to the 20th and 30th percentiles; Table S6: G2SFCA accessibility score comparison; Table S7: Population-to-facility ratio comparison; Table S8: Socioeconomic status comparison; Table S9: Age distribution comparison; Table S10: Race and ethnicity distribution comparison. USDA’s Metrics—Urban and Rural Section: Table S11: G2SFCA score, population-to-facility ratio, and socioeconomic status—USDA (Urban and Rural); Table S12: Age, race, and ethnicity distribution—USDA (Urban and Rural).
