# Supplementary material for: Identifying Areas with Low Access to the COVID-19 Vaccine: A New Objective Framework Incorporating Mobility Data
Source: Healthcare (Basel). 2025 Jun 6;13(12):1368. doi: 10.3390/healthcare13121368 (PMC12192818; doi:10.3390/healthcare13121368)
Supplement: Supplementary file 1 [file healthcare-13-01368-s001.zip › healthcare-3623444-supplementary.pdf]

healthcare

Supplementary materials

Table S1. Demographic Data Collected from United States Census Bureau.

Table Name	Level
B01003 ǀ TOTAL POPULATION	Block Groups
B06009 ǀ PLACE OF BIRTH BY EDUCATIONAL ATTAINMENT IN THE UNITED STATES	Census Tracts
S2301 ǀ EMPLOYMENT STATUS	Census Tracts
B19301 ǀ PER CAPITA INCOME IN THE PAST 12 MONTHS (IN 2019 INFLATION-ADJUSTED DOLLARS)	Census Tracts
B19301A ǀ PER CAPITA INCOME IN THE PAST 12 MONTHS (IN 2019 INFLATION-ADJUSTED DOLLARS) (WHITE ALONE)	Census Tracts
S2701 ǀ SELECTED CHARACTERISTICS OF HEALTH INSURANCE COVERAGE IN THE UNITED STATES	Census Tracts
S1701 ǀ POVERTY STATUS IN THE PAST 12 MONTHS	Census Tracts
S0101 ǀ AGE AND SEX	Census Tracts
B02001 ǀ RACE	Census Tracts
B01001 ǀ SEX BY AGE (HISPANIC OR LATINO)	Census Tracts

Table S2. U.S. Department of Agriculture Rural-Urban Commuting Area (RUCA) code:.

RUCA Code	Category
1	Metropolitan area core
2	Metropolitan area high commuting
3	Metropolitan area low commuting
4	Micropolitan area core
5	Micropolitan high commuting
6	Micropolitan low commuting
7	Small town core
8	Small town high commuting
9	Small town low commuting
10	Rural areas

Table S3. USDA Low-access Definition (Food).

Approach	Definition	Options (If any)
1	More than 500 people or 33% of the population live further than a pre-specified threshold distance from the nearest supermarket.	0.80 km for urban areas and 16.09 km for rural areas
		1.61 km for urban areas and 16.09 km for rural areas
		1.61 km for urban areas and 32.19 km for rural areas
2	At least 100 households living further than 0.80 km from the nearest supermarket have no	None

access to a vehicle or more than 500 people or 33% population live further than 32.19 km from the nearest supermarket.

Spatial Evaluation Metrics

A. Gaussian 2SFCA accessibility score

The G2SFCA score utilizes an extension to the 2SFCA method incorporating a Gaussian distance decay function to account for the negative correlation between distance and access (i.e., as distance from a resource increases, even within a catchment zone, access decreases). In this approach, the first step is to calculate a weighted supply-to-demand ratio R_f for each vaccination provider f using:

$$R_f = \frac{C_f}{\sum_{p \in \{t_{pf} \le t_0\}} P_p G\left(d_{pf}, d_0\right)}$$

where P_p is the population of block group p, C_f is the maximum supply of provider f (i.e., the maximum amount of vaccines provider f can administered in a single day), and $G\left(d_{pf}, d_0\right)$ is the Gaussian distance decay function given as:

$$G\left(d_{pf}, d_0\right) = \begin{cases} \dfrac{e^{-\frac{1}{2} \times \left(d_{pf}/d_0\right)^2} - e^{-\frac{1}{2}}}{1 - e^{-\frac{1}{2}}} & if\ d_{pf} \le d_0 \\ 0 & if\ d_{pf} > d_0 \end{cases}$$

where d_0 is the threshold distance or threshold travel time and d_{pf} is the distance or travel time between block group p and provider f. We utilize the travel time by car between each population (block group) and vaccine provider. For this analysis, we assume the supply capacity (C_f) of each provider is the same at 32 shots per day. For any block group p, we calculate the G2SFCA accessibility score, denoted A_p, using the following formula:

$$A_p = \sum_{f \in \{d_{pf} \le d_0\}} R_f\, G\left(d_{pf}, d_0\right)$$

Finally, we determine the G2SFCA accessibility score of each census tract, denoted by A_c, as the population-weighted accessibility of each block group within that tract:

$$A_c = \sum_{p \in B_c} A_p \frac{P_p}{\sum_{p \in B_c} P_p}$$

where B_c is the set of block groups that make up census tract c.

Consider, for example, a census tract c that consists of three block groups BG1, BG2, and BG3 and three vaccine providers F1, F2, and F3 provided in Figure S1. Observe that provider F1 is in the catchment zone of block group 1, provider F2 is in the catchment zones of block groups 1 and 2, and provider F3 is outside of the catchment zones of all block groups. Suppose the threshold travel time of block groups is 20 minutes.

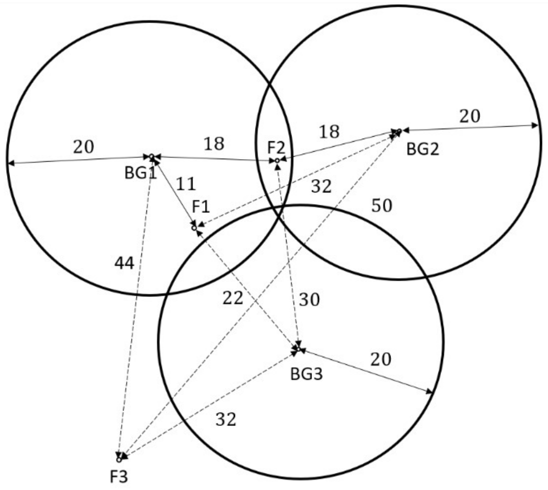

Figure S1. G2SFCA accessibility calculations example. The points labeled BG1, BG2, and BG3 represent the centroid location for block groups 1, 2, and 3, respectively. The points labeled F1, F2, and F3 represent the location of vaccination providers 1, 2, and 3, respectively. Travel time between centroid locations of block groups and providers within the corresponding catchment zones are marked with solid arrows. Travel time between centroid locations of block groups and providers outside of catchment zones are marked with dashed arrows.

Let's now assume that the populations for BG1, BG2 and BG3 are 400, 500, and 1,000, respectively. The first step is to calculate the weighted supply-to-demand ratio R_f for each vaccination provider F1, F2, and F3:

$$R_{F1} = \frac{C_{F1}}{P_{BG1}G(11,20)} = \frac{32}{(400)(0.64325)} = 0.12437$$

$$R_{F2} = \frac{C_{F2}}{P_{BG1}G(18,20) + P_{BG2}G(18,20)} = \frac{32}{(400)(0.15362) + (500)(0.15362)} = 0.23145$$

Where

$$G(11,20) = \frac{e^{-\frac{1}{2}\times(11/20)^2} - e^{-\frac{1}{2}}}{1-e^{-\frac{1}{2}}} = 0.64325; \qquad G(18,20) = \frac{e^{-\frac{1}{2}\times(18/20)^2} - e^{-\frac{1}{2}}}{1-e^{-\frac{1}{2}}} = 0.15362$$

and

$$G(22,20) = G(30,20) = G(32,20) = G(44,20) = G(50,20) = 0$$

Then, we calculate the accessibility of each block group as:

$$A_{BG1} = R_{F1}G(11,20) + R_{F2}G(18,20) = (0.12437)(0.64325) + (0.23145)(0.15362) = 0.11556$$
$$A_{BG2} = R_{F2}G(18,20) = (0.23145)(0.15362) = 0.03556$$
$$A_{BG3} = 0$$

Finally, the accessibility of this census tract is calculated as:

$$A_c = \frac{A_{BG1}P_{BG1} + A_{BG2}P_{BG2} + A_{BG1}P_{BG3}}{P_{BG1} + P_{BG2} + P_{BG3}} = \frac{(0.11556)(400) + (0.03556)(500) + 0(1000)}{400 + 500 + 1000}$$
$$= 0.03369$$

B. Population-to-facility ratio

For any census tract c, let F_p^c be the set of providers within the threshold travel time of block group $p \in B_c$. We calculate a population-to-facility ratio for census tract c, denoted as PFR_c, by dividing the total population in that census tract by the number of providers within the threshold travel time of each block group in that census tract:

$$PFR_c = \frac{\sum_{p \in B_c} P_p}{\sum_{p \in B_c} |F_p^c|}.$$

For instance, consider a census tract c that has been identified as a low-access census tract. Suppose that this census tract consists of two block groups, BG1 and BG2, with 700 and 800 people in it. Moreover, there are four and six providers within the threshold travel time of BG1 and BG2, respectively. The population-to-facility ratio of each census tract is calculated as:

$$PFR_c = \frac{700 + 800}{4 + 6} = 150$$

This value can be interpreted that, on average, there are 150 people being served per provider within that census tract. Note that if $\sum_{p \in B_c} |F_p^c| = 0$ (i.e., there are no facilities within any block group $p \in B_c$), then we set PFR_c to be the maximum population-to-facility ratio across all low-access census tracts that have at least one block group with a facility within the threshold distance. Namely, $PFR_c = \max_{\{c \,:\, F_p^c \neq \emptyset\}} PFR_c$.

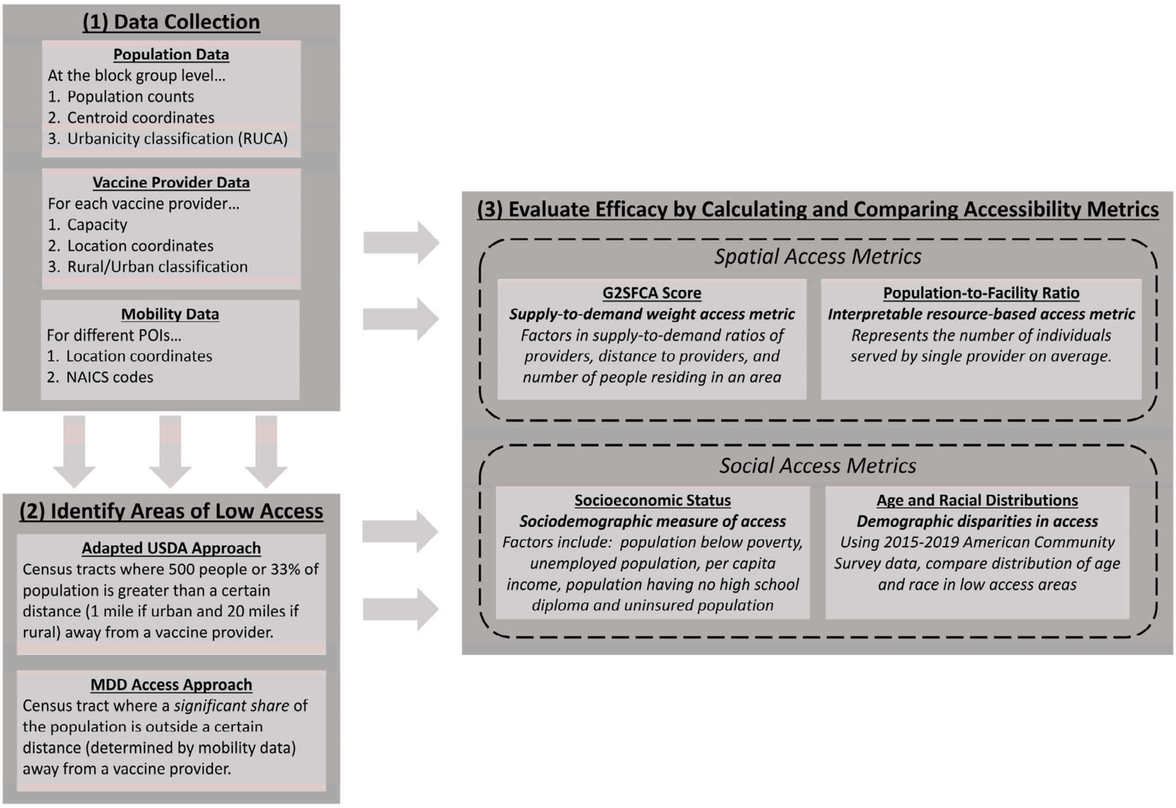

Figure S2. Overview of the research process. The steps in the process include (1) gathering and description of the data sources, (2) identification of "low access" areas using our proposed MDD approach and an adapted standard approach, and (3) evaluation of the proposed methods through the comparison of different measures of access.

Sensitivity Analysis

Note: "All" represents the situation that a census tract will be considered to be low access as long as no access block groups exist in that tract.

Table S4. Significant share determined by percentile.

Percentile	Significant Share
All	0.00%
5%	10.60%
10%	13.13%
15%	15.00%
20%	18.08%
25%	20.84%
30%	23.14%

Table S5. Rural and Urban Distribution of Low-access census tracts. The "USDA" bar represents the distribution of the identified census tracts (N) by the adapted United States Department of Agriculture definition of low food access. The "MDD" bars represent the distribution of the census tracts identified through the proposed mobility data driven definition across different seasons: Spring (March – May), Summer (June – August), Fall (September – November), and Winter (December – February).

Definition	Percentile	Urban	Rural	Total
USDA	-	255	17	272
MDD-Spring	All	14	39	53
	5%	12	38	50
	10%	12	36	48
	15%	12	33	45
	20%	12	30	42
	25%	12	29	41
	30%	11	28	39
MDD-Summer	All	9	30	39
	5%	8	30	38
	10%	8	28	36
	15%	8	26	34
	20%	8	23	31
	25%	8	21	29
	30%	7	20	27
MDD-Fall	All	14	39	53
	5%	12	38	50
	10%	12	36	48
	15%	12	33	45
	20%	12	30	42
	25%	12	29	41
	30%	11	28	39
NDD-Winter	All	14	48	62
	5%	12	46	58
	10%	12	43	55
	15%	12	41	53
	20%	12	39	51
	25%	12	38	50
	30%	11	37	48

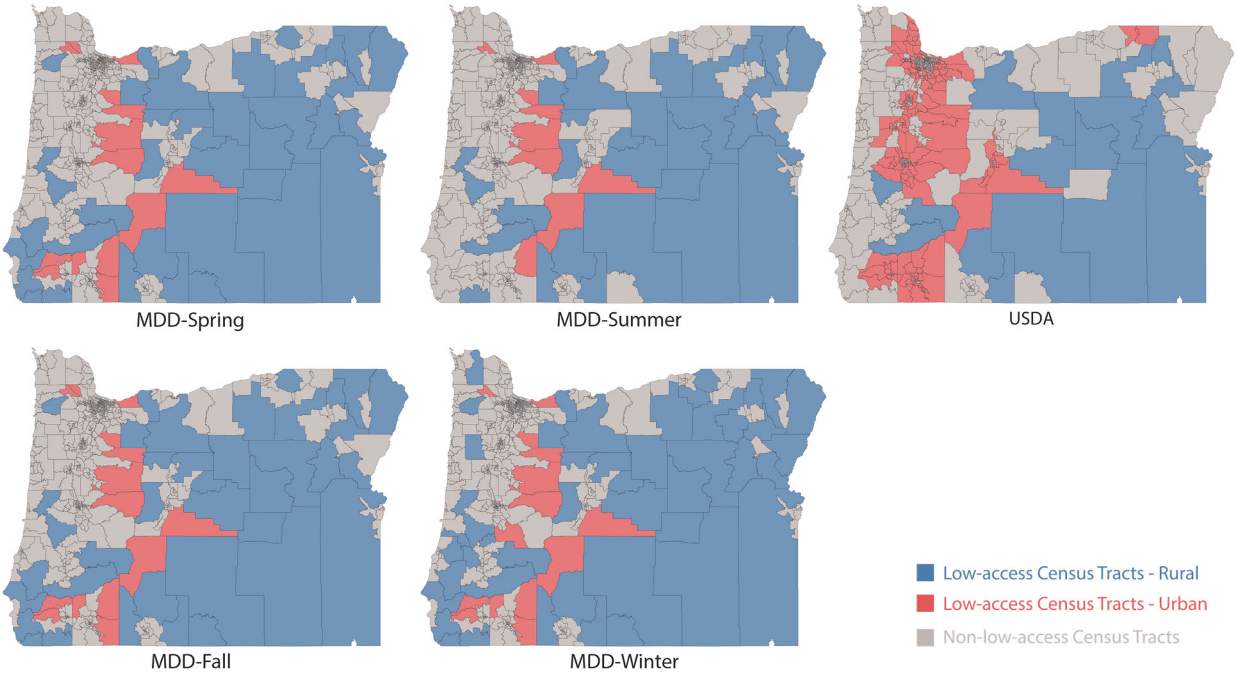

Figure S3. Geographic distribution of identified low-access census tracts across seasons ("All") with comparison of USDA in Oregon.

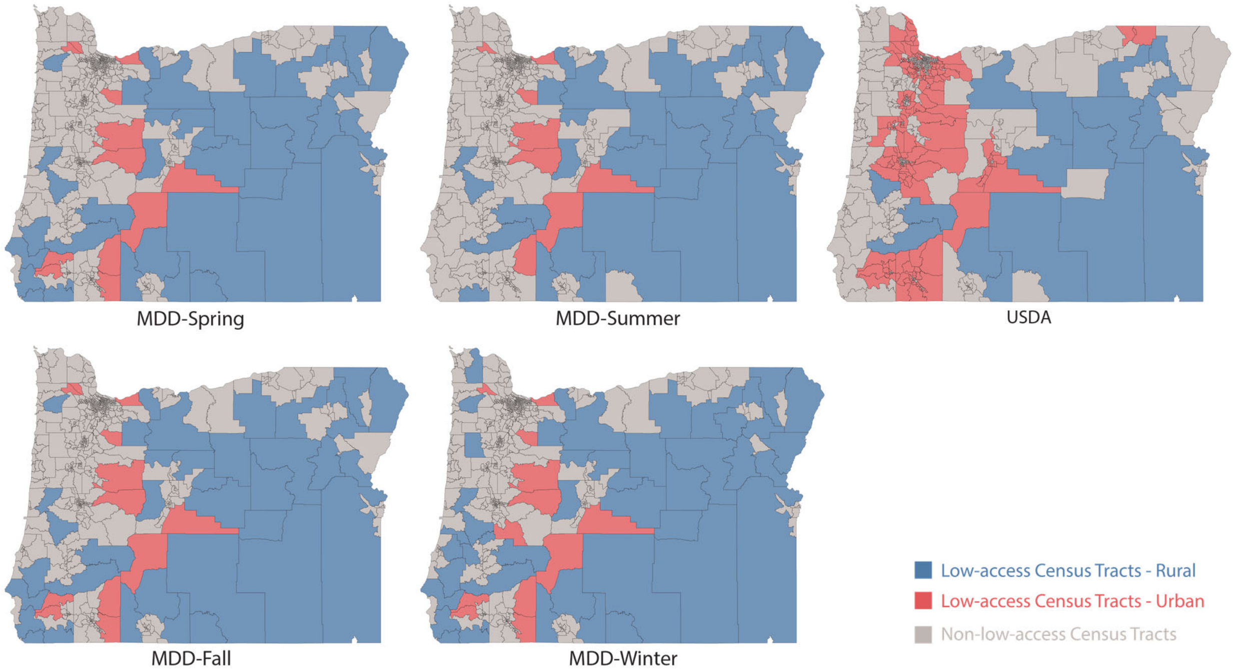

Figure S4. Geographic distribution of identified low-access census tracts across seasons (Γ = 5%) with comparison of USDA in Oregon.

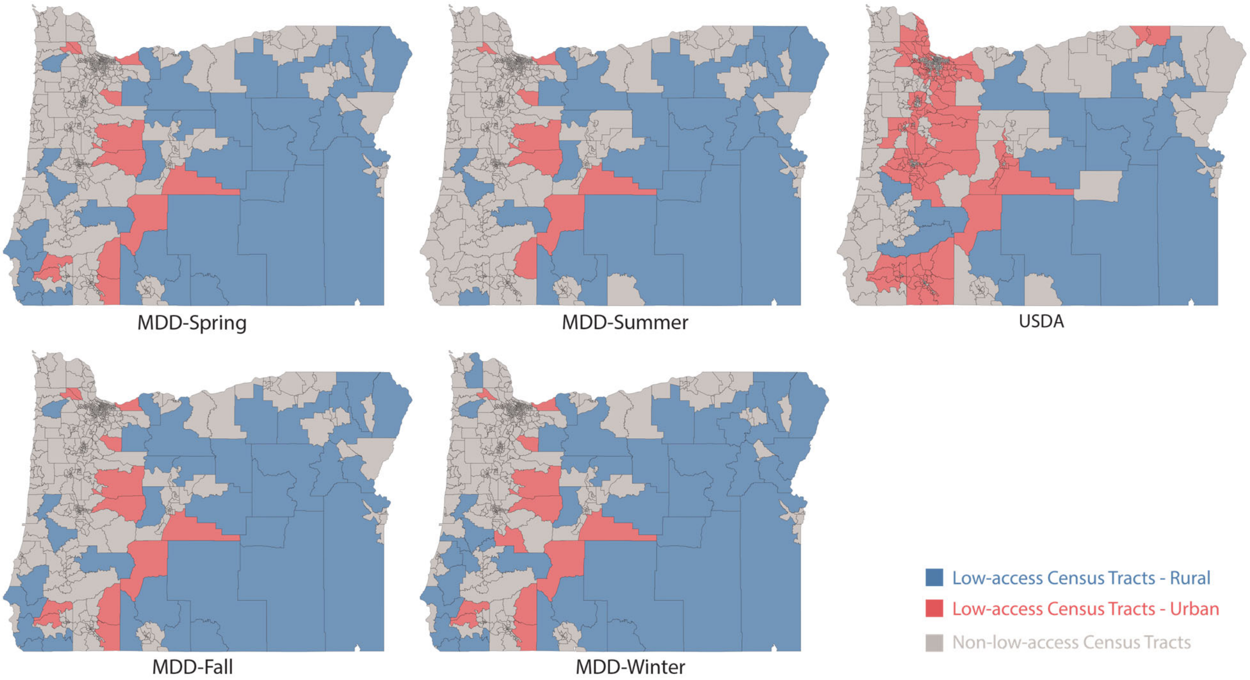

Figure S5. Geographic distribution of identified low-access census tracts across seasons (**Γ** = 10%) with comparison of USDA in Oregon.

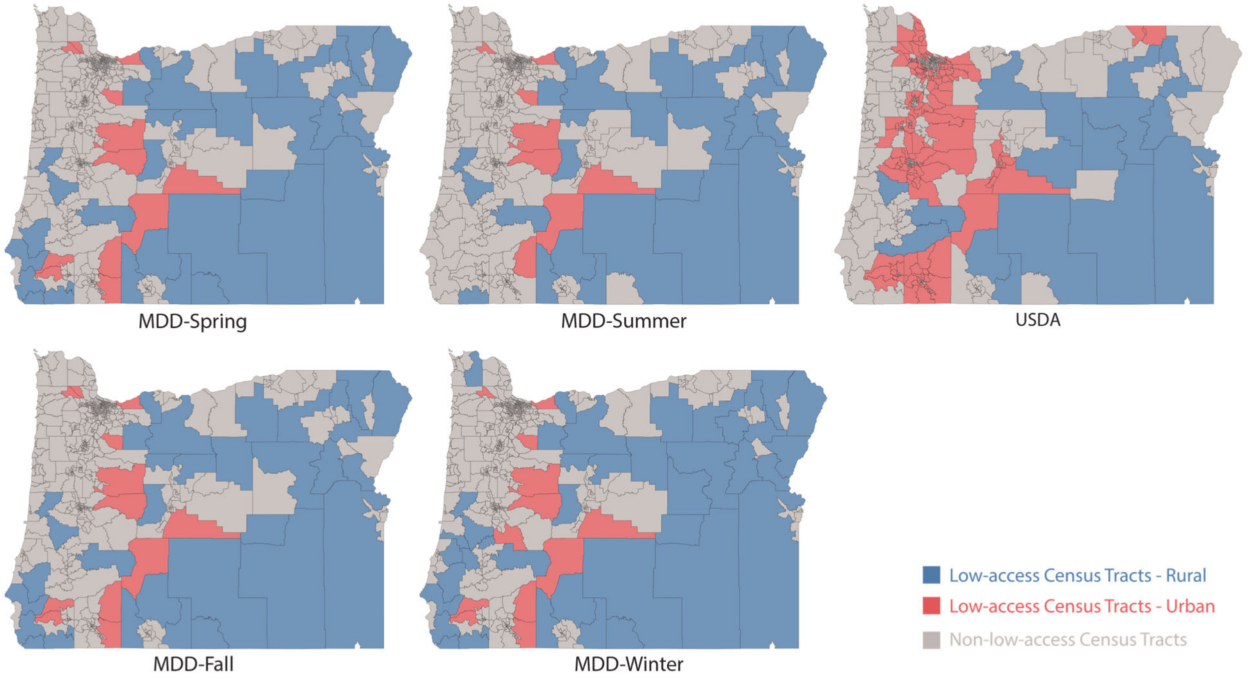

Figure S6. Geographic distribution of identified low-access census tracts across seasons (**Γ** = 15%) with comparison of USDA in Oregon.

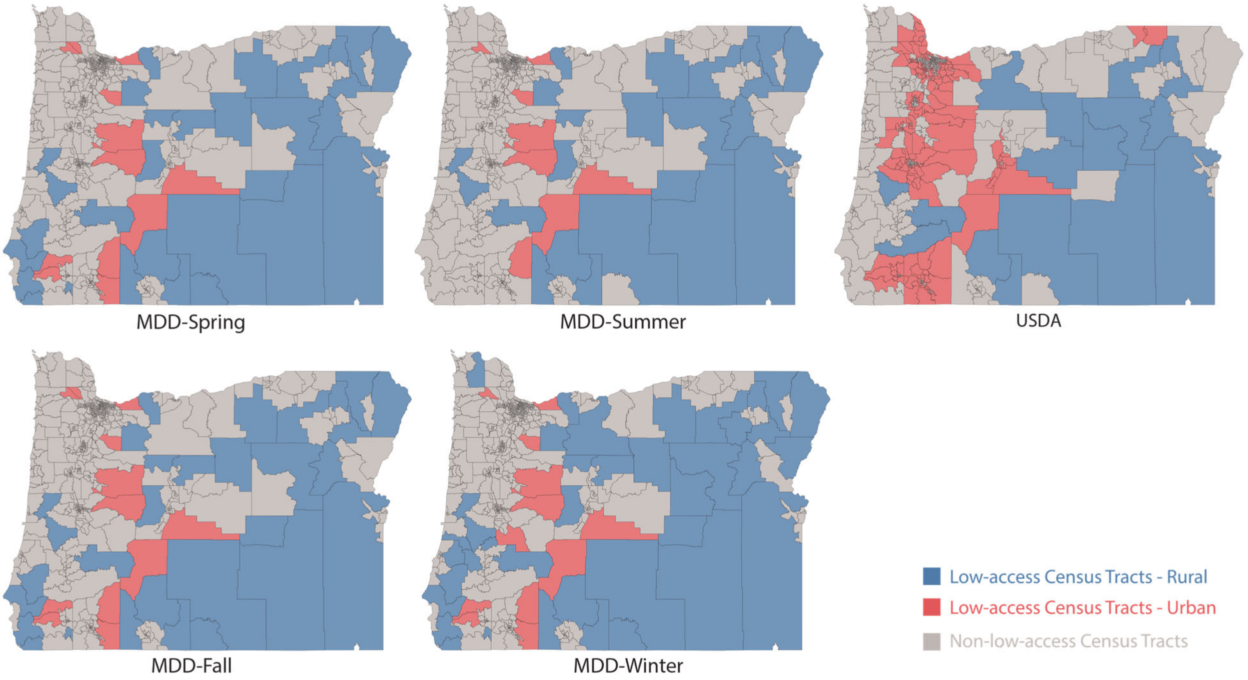

Figure S7. Geographic distribution of identified low-access census tracts across seasons (**Γ** = 20%) with comparison of USDA in Oregon.

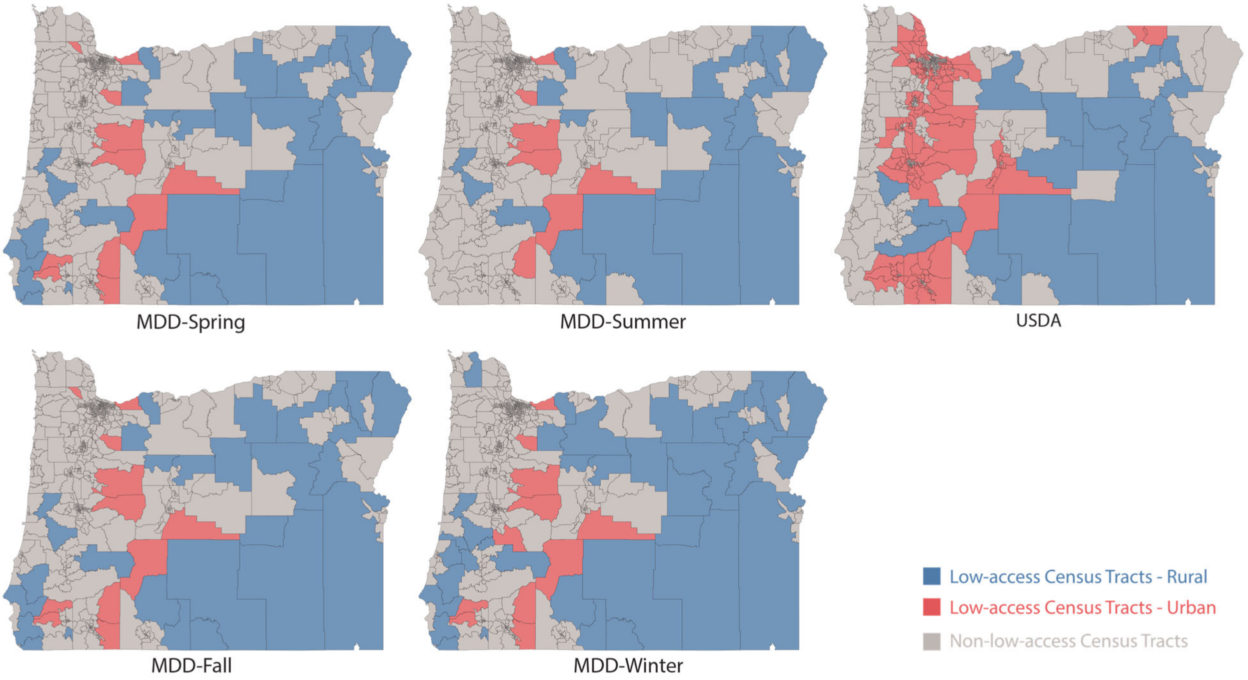

Figure S8. Geographic distribution of identified low-access census tracts across seasons (**Γ** = 30%) with comparison of USDA in Oregon.

Table S6. G2SFCA accessibility score comparison.

**Metrics**			**G2SFCA score**							
			20	30	40	50	60	70	80	90
**USDA**		**Median**	0.242	0.254	0.287	0.315	0.326	0.332	0.341	0.347
		IQR	0.226	0.240	0.231	0.218	0.193	0.164	0.133	0.107
MDD-Spring	All	Median	**0.000**	**0.040**	**0.048**	**0.070**	**0.092**	**0.107**	**0.126**	**0.156**
		IQR	0.143	0.187	0.158	0.148	0.156	0.154	0.162	0.152

	5%	Median	**0.000**	**0.027**	**0.045**	**0.069**	**0.089**	**0.106**	**0.125**	**0.153**
		IQR	0.133	0.178	0.154	0.154	0.169	0.160	0.163	0.157
	10%	Median	**0.000**	**0.016**	**0.044**	**0.065**	**0.085**	**0.100**	**0.124**	**0.144**
		IQR	0.087	0.151	0.146	0.154	0.169	0.160	0.162	0.156
	15%	Median	**0.000**	**0.012**	**0.042**	**0.062**	**0.082**	**0.094**	**0.121**	**0.138**
		IQR	0.081	0.130	0.126	0.137	0.151	0.155	0.143	0.151
	20%	Median	**0.000**	**0.008**	**0.038**	**0.055**	**0.080**	**0.093**	**0.117**	**0.144**
		IQR	0.070	0.126	0.135	0.135	0.151	0.165	0.164	0.156
	25%	Median	**0.000**	**0.003**	**0.037**	**0.054**	**0.077**	**0.092**	**0.121**	**0.150**
		IQR	0.081	0.130	0.142	0.137	0.151	0.174	0.171	0.157
	30%	Median	**0.000**	**0.000**	**0.019**	**0.045**	**0.063**	**0.088**	**0.112**	**0.138**
		IQR	0.059	0.123	0.127	0.132	0.150	0.160	0.166	0.152
MDD-Summer	All	Median	**0.000**	**0.020**	**0.045**	**0.062**	**0.077**	**0.092**	**0.112**	**0.117**
		IQR	0.026	0.114	0.108	0.125	0.150	0.144	0.142	0.153
	5%	Median	**0.000**	**0.016**	**0.044**	**0.059**	**0.080**	**0.093**	**0.117**	**0.126**
		IQR	0.025	0.115	0.110	0.126	0.150	0.149	0.143	0.155
	10%	Median	**0.000**	**0.008**	**0.043**	**0.055**	**0.076**	**0.090**	**0.106**	**0.113**
		IQR	0.013	0.094	0.107	0.124	0.149	0.142	0.145	0.145
	15%	Median	**0.000**	**0.004**	**0.041**	**0.050**	**0.069**	**0.086**	**0.100**	**0.108**
		IQR	0.020	0.081	0.102	0.123	0.140	0.138	0.132	0.113
	20%	Median	**0.000**	**0.000**	**0.019**	**0.034**	**0.056**	**0.085**	**0.099**	**0.108**
		IQR	0.016	0.067	0.097	0.101	0.128	0.138	0.151	0.134
	25%	Median	**0.000**	**0.000**	**0.019**	**0.033**	**0.044**	**0.064**	**0.086**	**0.086**
		IQR	0.023	0.088	0.089	0.080	0.107	0.132	0.150	0.125
	30%	Median	**0.000**	**0.000**	**0.013**	**0.022**	**0.041**	**0.052**	**0.072**	**0.082**
		IQR	0.016	0.067	0.077	0.079	0.096	0.122	0.130	0.117
MDD-Fall	All	Median	**0.000**	**0.040**	**0.048**	**0.070**	**0.092**	**0.107**	**0.126**	**0.156**
		IQR	0.143	0.187	0.158	0.148	0.156	0.154	0.162	0.152
	5%	Median	**0.000**	**0.027**	**0.045**	**0.069**	**0.089**	**0.106**	**0.125**	**0.153**
		IQR	0.133	0.178	0.154	0.154	0.169	0.160	0.163	0.157
	10%	Median	**0.000**	**0.016**	**0.044**	**0.065**	**0.085**	**0.100**	**0.124**	**0.144**
		IQR	0.087	0.151	0.146	0.154	0.169	0.160	0.162	0.156
	15%	Median	**0.000**	**0.012**	**0.042**	**0.062**	**0.082**	**0.094**	**0.121**	**0.138**
		IQR	0.081	0.130	0.126	0.137	0.151	0.155	0.143	0.151
	20%	Median	**0.000**	**0.008**	**0.038**	**0.055**	**0.080**	**0.093**	**0.117**	**0.144**
		IQR	0.070	0.126	0.135	0.135	0.151	0.165	0.164	0.156
	25%	Median	**0.000**	**0.003**	**0.037**	**0.054**	**0.077**	**0.092**	**0.121**	**0.150**
		IQR	0.081	0.130	0.142	0.137	0.151	0.174	0.171	0.157
	30%	Median	**0.000**	**0.000**	**0.019**	**0.045**	**0.063**	**0.088**	**0.112**	**0.138**
		IQR	0.059	0.123	0.127	0.132	0.150	0.160	0.166	0.152
MDD-Winter	All	Median	**0.000**	**0.041**	**0.058**	**0.077**	**0.101**	**0.118**	**0.145**	**0.163**
		IQR	0.189	0.197	0.180	0.179	0.185	0.180	0.139	0.146
	5%	Median	**0.000**	**0.037**	**0.047**	**0.074**	**0.098**	**0.111**	**0.138**	**0.161**
		IQR	0.176	0.188	0.168	0.174	0.165	0.160	0.159	0.142
	10%	Median	**0.000**	**0.020**	**0.045**	**0.070**	**0.092**	**0.107**	**0.126**	**0.156**
		IQR	0.171	0.188	0.167	0.178	0.172	0.165	0.163	0.146
	15%	Median	**0.000**	**0.012**	**0.043**	**0.068**	**0.086**	**0.106**	**0.124**	**0.150**
		IQR	0.161	0.187	0.165	0.180	0.177	0.167	0.165	0.149
	20%	Median	**0.000**	**0.006**	**0.042**	**0.062**	**0.086**	**0.107**	**0.126**	**0.156**
		IQR	0.133	0.185	0.167	0.184	0.182	0.174	0.166	0.155
	25%	Median	**0.000**	**0.005**	**0.041**	**0.062**	**0.089**	**0.109**	**0.129**	**0.159**
		IQR	0.147	0.186	0.169	0.185	0.184	0.178	0.167	0.159
	30%	Median	**0.000**	**0.004**	**0.036**	**0.062**	**0.086**	**0.106**	**0.125**	**0.153**
		IQR	0.118	0.184	0.170	0.188	0.184	0.177	0.167	0.157

Table S7. Population-to-Facility ratio comparison.

**Metrics**			**Population-to-Facility ratio**							
			20	30	40	50	60	70	80	90
**USDA**		**Median**	50	23	14	11	8	6	5	4
		IQR	243	65	31	20	15	12	10	9
MDD-Spring	All	Median	**4954**	**822**	**383**	**212**	**132**	**96**	**69**	**48**
		IQR	4133	4668	2295	978	495	269	188	143
	5%	Median	**4954**	**1075**	**434**	**240**	**143**	**111**	**75**	**53**
		IQR	4123	4652	2271	1365	568	285	207	161
	10%	Median	**4954**	**1223**	**450**	**245**	**150**	**118**	**77**	**58**
		IQR	4104	4650	2247	1489	1241	306	212	172
	15%	Median	**4954**	**1415**	**449**	**244**	**150**	**117**	**78**	**60**
		IQR	4095	4653	2258	1498	3176	415	212	166
	20%	Median	**4954**	**3185**	**416**	**252**	**150**	**120**	**83**	**56**
		IQR	4123	4663	2271	1504	3179	793	282	209
	25%	Median	**4954**	**4954**	**449**	**260**	**151**	**122**	**91**	**60**
		IQR	4133	4653	2258	1498	3180	919	306	223
	30%	Median	**4954**	**4954**	**450**	**262**	**174**	**132**	**106**	**66**
		IQR	4161	4645	2240	1483	3170	1636	371	222
MDD-Summer	All	Median	**4954**	**1089**	**472**	**244**	**137**	**105**	**74**	**52**
		IQR	3637	4657	2303	1527	1905	373	274	182
	5%	Median	**4954**	**1252**	**478**	**252**	**143**	**111**	**75**	**53**
		IQR	3665	4647	2293	1517	2531	399	289	184
	10%	Median	**4954**	**1435**	**535**	**295**	**165**	**118**	**77**	**58**
		IQR	3578	4644	2281	1491	3167	526	323	196
	15%	Median	**4954**	**3205**	**478**	**295**	**143**	**111**	**77**	**57**
		IQR	3655	4647	2290	1501	3170	768	405	211
	20%	Median	**4954**	**4954**	**472**	**330**	**150**	**117**	**75**	**52**
		IQR	3677	4657	2303	1513	3174	1650	448	266
	25%	Median	**4954**	**4954**	**472**	**413**	**181**	**122**	**106**	**66**
		IQR	3815	4648	2295	1506	3176	2417	451	305
	30%	Median	**4954**	**4954**	**822**	**449**	**225**	**132**	**108**	**71**
		IQR	3974	4633	2267	1483	3162	2375	835	297
MDD-Fall	All	Median	**4954**	**822**	**383**	**212**	**132**	**96**	**69**	**48**
		IQR	4133	4668	2295	978	495	269	188	143
	5%	Median	**4954**	**1075**	**434**	**240**	**143**	**111**	**75**	**53**
		IQR	4123	4652	2271	1365	568	285	207	161
	10%	Median	**4954**	**1223**	**450**	**245**	**150**	**118**	**77**	**58**
		IQR	4104	4650	2247	1489	1241	306	212	172
	15%	Median	**4954**	**1415**	**449**	**244**	**150**	**117**	**78**	**60**
		IQR	4095	4653	2258	1498	3176	415	212	166
	20%	Median	**4954**	**3185**	**416**	**252**	**150**	**120**	**83**	**56**
		IQR	4123	4663	2271	1504	3179	793	282	209
	25%	Median	**4954**	**4954**	**449**	**260**	**151**	**122**	**91**	**60**
		IQR	4133	4653	2258	1498	3180	919	306	223
	30%	Median	**4954**	**4954**	**450**	**262**	**174**	**132**	**106**	**66**
		IQR	4161	4645	2240	1483	3170	1636	371	222
MDD-Winter	All	Median	**4954**	**670**	**345**	**189**	**117**	**89**	**57**	**42**
		IQR	4395	4675	1414	669	400	205	160	136
	5%	Median	**4954**	**940**	**402**	**211**	**128**	**96**	**61**	**45**
		IQR	4267	4652	2085	913	459	247	182	146
	10%	Median	**4954**	**1061**	**449**	**237**	**137**	**105**	**69**	**48**
		IQR	4161	4645	2286	946	532	267	197	154
	15%	Median	**4954**	**1089**	**449**	**237**	**137**	**105**	**74**	**48**

	IQR	4133	4648	2295	952	604	288	212	166
20%	Median	**4954**	**1358**	**449**	**244**	**137**	**105**	**74**	**48**
	IQR	4161	4651	2300	1236	799	362	227	181
25%	Median	**4954**	**1386**	**450**	**252**	**143**	**111**	**75**	**50**
	IQR	4176	4647	2290	1365	896	399	234	189
30%	Median	**4954**	**1435**	**461**	**261**	**150**	**120**	**77**	**56**
	IQR	4249	4639	2263	1487	1536	545	252	194

Table S8. Socioeconomic Status comparison.

Metrics			Socioeconomic Status				
			Poverty Rate	No High School Diploma Rate	Unemployment Rate	Uninsured Rate	Per Capita Income
**USDA**		**Median**	9.65%	7.59%	4.90%	5.65%	33933
		IQR	8.00%	6.70%	3.60%	4.10%	13872
MDD-Spring	All	Median	**13.30%**	**9.15%**	**6.80%**	**6.50%**	**27575**
		IQR	8.20%	5.38%	5.20%	2.80%	6697
	5%	Median	**13.30%**	**9.17%**	**7.00%**	**6.50%**	**27231**
		IQR	8.25%	4.96%	5.30%	3.15%	6681
	10%	Median	**13.30%**	**9.17%**	**7.00%**	**6.50%**	**27231**
		IQR	8.30%	4.78%	5.10%	2.95%	6749
	15%	Median	**13.30%**	**8.77%**	**6.80%**	**6.50%**	**27592**
		IQR	8.20%	4.28%	4.60%	2.80%	6960
	20%	Median	**13.30%**	**8.66%**	**6.80%**	**6.50%**	**28154**
		IQR	8.18%	4.31%	4.58%	2.83%	7537
	25%	Median	**13.30%**	**8.56%**	**6.80%**	**6.50%**	**27592**
		IQR	8.20%	4.29%	4.60%	2.80%	7744
	30%	Median	**14.00%**	**8.77%**	**7.20%**	**6.50%**	**26837**
		IQR	8.25%	4.14%	4.80%	3.00%	7059
MDD-Summer	All	Median	**12.50%**	**9.10%**	**6.70%**	**6.50%**	**27993**
		IQR	7.15%	4.57%	5.45%	3.10%	6905
	5%	Median	**12.75%**	**9.13%**	**6.65%**	**6.50%**	**27793**
		IQR	7.18%	4.60%	5.05%	3.13%	6788
	10%	Median	**12.75%**	**8.96%**	**6.65%**	**6.45%**	**28154**
		IQR	7.28%	4.43%	4.95%	2.95%	6858
	15%	Median	**12.75%**	**8.65%**	**6.15%**	**6.50%**	**28718**
		IQR	7.13%	4.31%	4.75%	3.13%	7449
	20%	Median	**11.70%**	**8.41%**	**5.70%**	**6.50%**	**28719**
		IQR	7.25%	3.99%	4.65%	3.10%	7921
	25%	Median	**13.00%**	**8.39%**	**6.60%**	**6.50%**	**28716**
		IQR	7.10%	3.69%	4.80%	3.10%	8495
	30%	Median	**14.00%**	**8.41%**	**6.70%**	**6.50%**	**27592**
		IQR	6.20%	3.93%	4.65%	3.50%	7596
MDD-Fall	All	Median	**13.30%**	**9.15%**	**6.80%**	**6.50%**	**27575**
		IQR	8.20%	5.38%	5.20%	2.80%	6697
	5%	Median	**13.30%**	**9.17%**	**7.00%**	**6.50%**	**27231**
		IQR	8.25%	4.96%	5.30%	3.15%	6681
	10%	Median	**13.30%**	**9.17%**	**7.00%**	**6.50%**	**27231**
		IQR	8.30%	4.78%	5.10%	2.95%	6749
	15%	Median	**13.30%**	**8.77%**	**6.80%**	**6.50%**	**27592**
		IQR	8.20%	4.28%	4.60%	2.80%	6960
	20%	Median	**13.30%**	**8.66%**	**6.80%**	**6.50%**	**28154**
		IQR	8.18%	4.31%	4.58%	2.83%	7537
	25%	Median	**13.30%**	**8.56%**	**6.80%**	**6.50%**	**27592**
		IQR	8.20%	4.29%	4.60%	2.80%	7744

	30%	Median	**14.00%**	**8.77%**	**7.20%**	**6.50%**	**26837**
		IQR	8.25%	4.14%	4.80%	3.00%	7059
MDD-Winter	All	Median	**13.30%**	**9.13%**	**6.80%**	**6.40%**	**27564**
		IQR	8.13%	5.34%	4.58%	2.68%	6649
	5%	Median	**13.50%**	**9.17%**	**6.95%**	**6.45%**	**27293**
		IQR	8.20%	5.29%	4.58%	2.73%	6649
	10%	Median	**13.30%**	**9.15%**	**6.80%**	**6.40%**	**27552**
		IQR	8.15%	5.16%	4.55%	2.65%	6793
	15%	Median	**13.70%**	**8.77%**	**6.80%**	**6.40%**	**27575**
		IQR	8.20%	4.87%	4.50%	2.60%	6916
	20%	Median	**13.30%**	**8.77%**	**6.80%**	**6.50%**	**27592**
		IQR	8.15%	4.77%	4.50%	2.75%	7043
	25%	Median	**13.50%**	**8.66%**	**6.80%**	**6.50%**	**27572**
		IQR	8.03%	4.60%	4.48%	2.73%	7283
	30%	Median	13.85%	8.77%	6.95%	6.50%	27227
		IQR	7.20%	4.69%	4.53%	2.85%	6709

Table S9. Age distribution comparison.

Metrics		Age			
		Under 5	5 to 19	20 to 64	65+
**Oregon**		5.58%	17.80%	59.43%	17.18%
USDA		5.49%	**18.51%**	57.29%	**18.71%**
MDD-Spring	0%	4.53%	15.29%	54.99%	**25.19%**
	5%	4.64%	15.56%	54.35%	**25.46%**
	10%	4.73%	15.49%	54.27%	**25.52%**
	15%	4.54%	15.46%	54.25%	**25.75%**
	20%	4.52%	15.53%	54.41%	**25.54%**
	25%	4.45%	15.60%	54.58%	**25.37%**
	30%	4.44%	15.18%	54.50%	**25.87%**
MDD-Summer	0%	4.61%	16.02%	54.30%	**25.08%**
	5%	4.63%	16.12%	54.19%	**25.06%**
	10%	4.76%	16.08%	54.06%	**25.10%**
	15%	4.77%	16.23%	54.23%	**24.77%**
	20%	4.78%	16.45%	54.45%	**24.33%**
	25%	4.66%	16.55%	54.70%	**24.09%**
	30%	4.68%	16.05%	54.60%	**24.66%**
MDD-Fall	0%	4.53%	15.29%	54.99%	**25.19%**
	5%	4.64%	15.56%	54.35%	**25.46%**
	10%	4.73%	15.49%	54.27%	**25.52%**
	15%	4.54%	15.46%	54.25%	**25.75%**
	20%	4.52%	15.53%	54.41%	**25.54%**
	25%	4.45%	15.60%	54.58%	**25.37%**
	30%	4.44%	15.18%	54.50%	**25.87%**
MDD-Winter	0%	4.69%	15.44%	54.74%	**25.13%**
	5%	4.76%	15.54%	54.24%	**25.46%**
	10%	4.79%	15.41%	54.02%	**25.77%**
	15%	4.63%	15.39%	53.97%	**26.01%**
	20%	4.62%	15.51%	54.27%	**25.60%**
	25%	4.56%	15.57%	54.41%	**25.47%**
	30%	4.56%	15.23%	54.33%	**25.88%**

Table S10. Race and ethnicity distribution comparison.

**Metrics**		Race							Ethnicity	
		White alone	Black or African American alone	American Indian and Alaska Native alone	Asian alone	Native Hawaiian and Other Pacific Islander alone	Some other race alone	Two or more races	Hispanic or Latino	Non-Hispanic or Latino
**Oregon**		84.29%	1.91%	1.16%	4.37%	0.40%	3.07%	4.80%	13.01%	86.99%
USDA		**86.51%**	1.24%	0.96%	4.01%	0.29%	2.52%	4.47%	11.25%	**88.75%**
MDD-Spring	0%	**89.84%**	0.68%	**3.72%**	0.72%	0.13%	1.18%	3.74%	7.22%	**92.78%**
	5%	**89.79%**	0.61%	**3.90%**	0.74%	0.13%	1.08%	3.75%	7.20%	**92.80%**
	10%	**89.54%**	0.65%	**4.10%**	0.76%	0.14%	1.09%	3.72%	7.09%	**92.91%**
	15%	**89.07%**	0.68%	**4.49%**	0.76%	0.14%	1.13%	3.73%	6.80%	**93.20%**
	20%	**88.90%**	0.69%	**4.41%**	0.77%	0.14%	1.24%	3.85%	7.12%	**92.88%**
	25%	**88.77%**	0.71%	**4.50%**	0.78%	0.14%	1.27%	3.84%	7.25%	**92.75%**
	30%	**88.62%**	0.76%	**4.89%**	0.77%	0.13%	1.09%	3.74%	7.16%	**92.84%**
MDD-Summer	0%	**89.50%**	0.61%	**4.65%**	0.71%	0.15%	1.10%	3.29%	6.70%	**93.30%**
	5%	**89.46%**	0.62%	**4.72%**	0.71%	0.15%	1.11%	3.23%	6.78%	**93.22%**
	10%	**89.09%**	0.68%	**5.07%**	0.74%	0.17%	1.13%	3.13%	6.58%	**93.42%**
	15%	**88.53%**	0.73%	**5.42%**	0.72%	0.17%	1.21%	3.22%	6.89%	**93.11%**
	20%	**88.21%**	0.75%	**5.44%**	0.72%	0.18%	1.38%	3.32%	7.33%	**92.67%**
	25%	**87.83%**	0.78%	**5.74%**	0.76%	0.14%	1.44%	3.31%	7.45%	**92.55%**
	30%	**87.47%**	0.87%	**6.50%**	0.74%	0.13%	1.21%	3.09%	7.33%	**92.67%**
MDD-Fall	0%	**89.84%**	0.68%	**3.72%**	0.72%	0.13%	1.18%	3.74%	7.22%	**92.78%**
	5%	**89.79%**	0.61%	**3.90%**	0.74%	0.13%	1.08%	3.75%	7.20%	**92.80%**
	10%	**89.54%**	0.65%	**4.10%**	0.76%	0.14%	1.09%	3.72%	7.09%	**92.91%**
	15%	**89.07%**	0.68%	**4.49%**	0.76%	0.14%	1.13%	3.73%	6.80%	**93.20%**
	20%	**88.90%**	0.69%	**4.41%**	0.77%	0.14%	1.24%	3.85%	7.12%	**92.88%**
	25%	**88.77%**	0.71%	**4.50%**	0.78%	0.14%	1.27%	3.84%	7.25%	**92.75%**
	30%	**88.62%**	0.76%	**4.89%**	0.77%	0.13%	1.09%	3.74%	7.16%	**92.84%**
MDD-Winter	0%	**89.89%**	0.64%	**3.67%**	0.68%	0.10%	1.09%	3.93%	6.99%	**93.01%**
	5%	**89.86%**	0.57%	**3.84%**	0.69%	0.10%	1.00%	3.94%	6.91%	**93.09%**
	10%	**89.87%**	0.58%	**3.82%**	0.68%	0.11%	1.03%	3.90%	6.88%	**93.12%**
	15%	**89.67%**	0.60%	**4.04%**	0.69%	0.11%	1.03%	3.86%	6.52%	**93.48%**
	20%	**89.32%**	0.61%	**4.22%**	0.68%	0.12%	1.09%	3.96%	6.66%	**93.34%**
	25%	**89.23%**	0.62%	**4.28%**	0.69%	0.11%	1.11%	3.95%	6.76%	**93.24%**
	30%	**89.14%**	0.66%	**4.58%**	0.68%	0.10%	0.96%	3.88%	6.64%	**93.36%**

USDA's Metrics – Urban and Rural

Table S11. G2SFCA Score, population-to-facility ratio *and* socioeconomic status – USDA (Urban and Rural).

**Metrics**		**USDA-All**		**USDA-Urban**		**USDA-Rural**	
		**Median**	**IQR**	**Median**	**IQR**	**Median**	**IQR**
**Calculated Metrics**	**Threshold Time - d0**						
**G2SFCA score**	**20**	0.242	0.226	0.252	0.201	0.000	0.023
	**30**	0.254	0.240	0.268	0.221	0.004	0.116
	**40**	0.287	0.231	0.301	0.218	0.039	0.079
	**50**	0.315	0.218	0.324	0.194	0.045	0.089
	**60**	0.326	0.193	0.333	0.172	0.082	0.083
	**70**	0.332	0.164	0.336	0.140	0.088	0.122
	**80**	0.341	0.133	0.345	0.109	0.099	0.140

	**90**	0.347	0.107	0.351	0.081	0.108	0.147
**Population-to-facility ratio**	**20**	50	243	47	151	4954	3539
	**30**	23	65	20	41	1455	4323
	**40**	14	31	13	21	485	2258
	**50**	11	20	10	15	397	895
	**60**	8	15	7	10	181	429
	**70**	6	12	6	9	132	202
	**80**	5	10	5	6	108	208
	**90**	4	9	4	5	71	137
**Non-calculated Metrics**	**Indicator**						
**Socioeconomic Status**	**Poverty Rate**	9.65%	8.00%	9.40%	7.35%	14.20%	6.70%
	**No High School Diploma Rate**	7.59%	6.70%	7.46%	6.61%	10.32%	3.14%
	**Unemployment Rate**	4.90%	3.60%	4.90%	3.60%	6.70%	5.20%
	**Uninsured Rate**	5.65%	4.10%	5.50%	4.25%	6.90%	3.00%
	**Per Capita Income**	33933	13872	34581	13699	26837	5231

Table S12. Age, race and ethnicity distribution – USDA (Urban and Rural).

Age or Race	USDA-All	USDA-Urban	USDA-Rural
**Age**			
Under 5	5.49%	5.54%	4.32%
5 to 19	18.51%	18.65%	14.81%
20 to 64	57.29%	57.40%	54.62%
65+	18.71%	18.41%	26.24%
**Race**			
White alone	86.51%	86.38%	90.01%
Black or African American alone	1.24%	1.26%	0.77%
American Indian and Alaska Native alone	0.96%	0.83%	4.11%
Asian alone	4.01%	4.14%	0.88%
Native Hawaiian and Other Pacific Islander alone	0.29%	0.30%	0.15%
Some other race alone	2.52%	2.58%	1.02%
Two or more races	4.47%	4.52%	3.06%
**Ethnicity**			
Hispanic or Latino	11.25%	11.45%	6.12%
Non-Hispanic or Latino	88.75%	88.55%	93.88%
